# Supplementary material for: A novel live-cell imaging assay reveals regulation of endosome maturation
Source: eLife. 2021 Nov 30;10:e70982. doi: 10.7554/eLife.70982 (PMC8635980; doi:10.7554/eLife.70982)
Supplement: Figure 12—figure supplement 2—source data 1. [file elife-70982-fig12-figsupp2-data1.pdf]

Originals

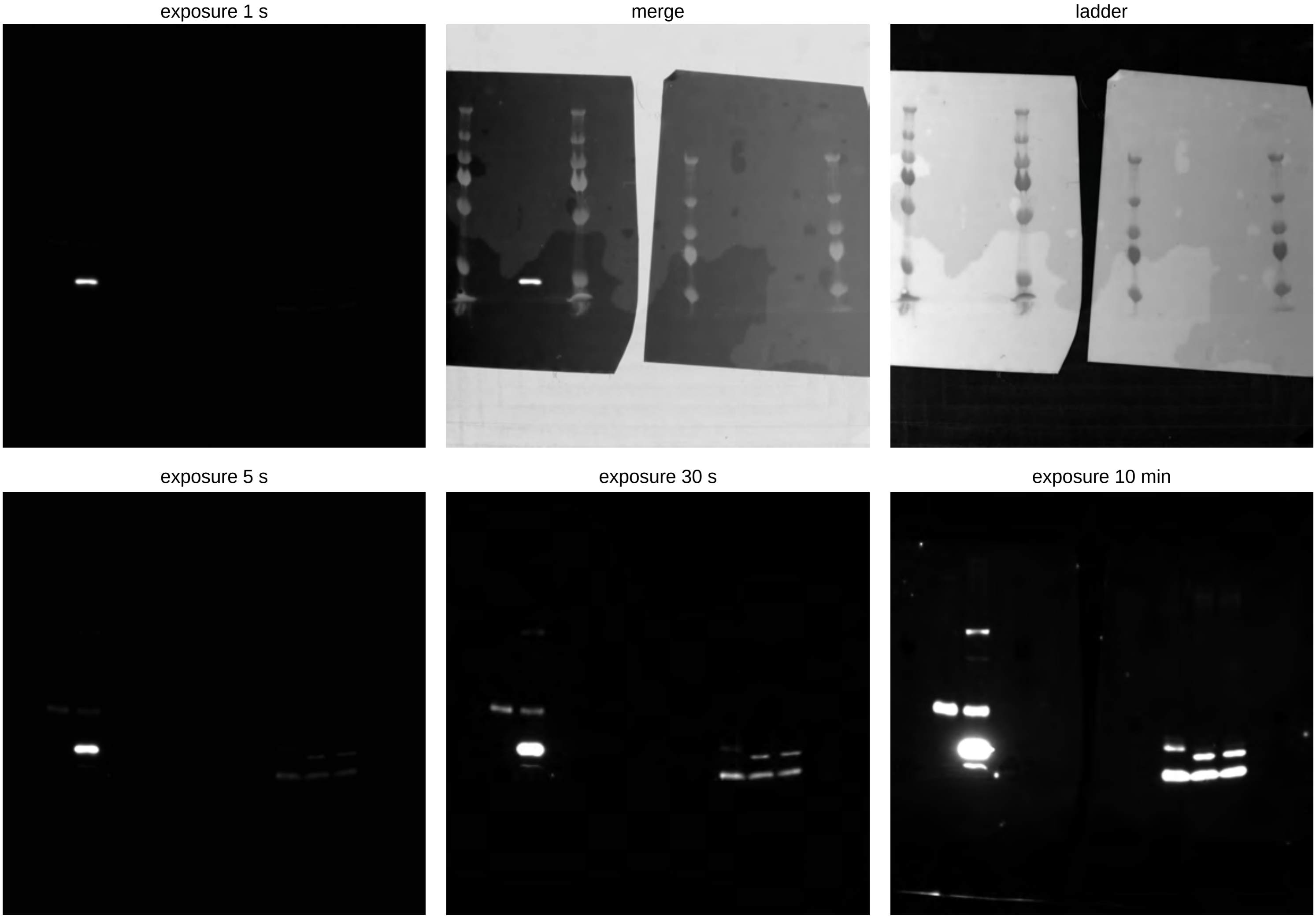

colour inverted

Annotated

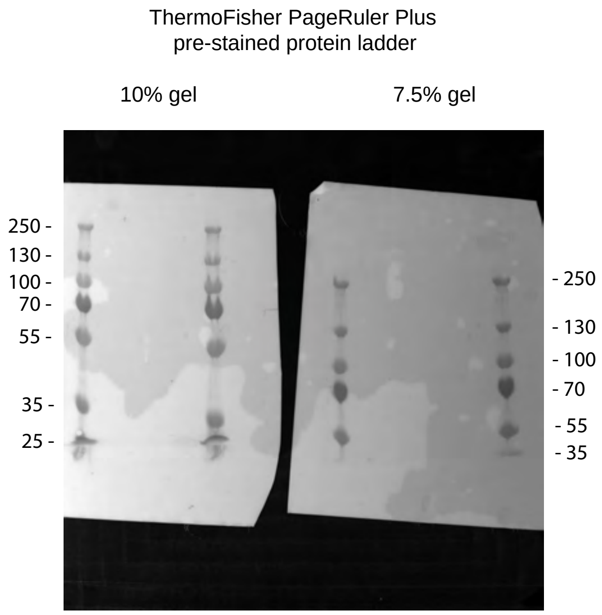

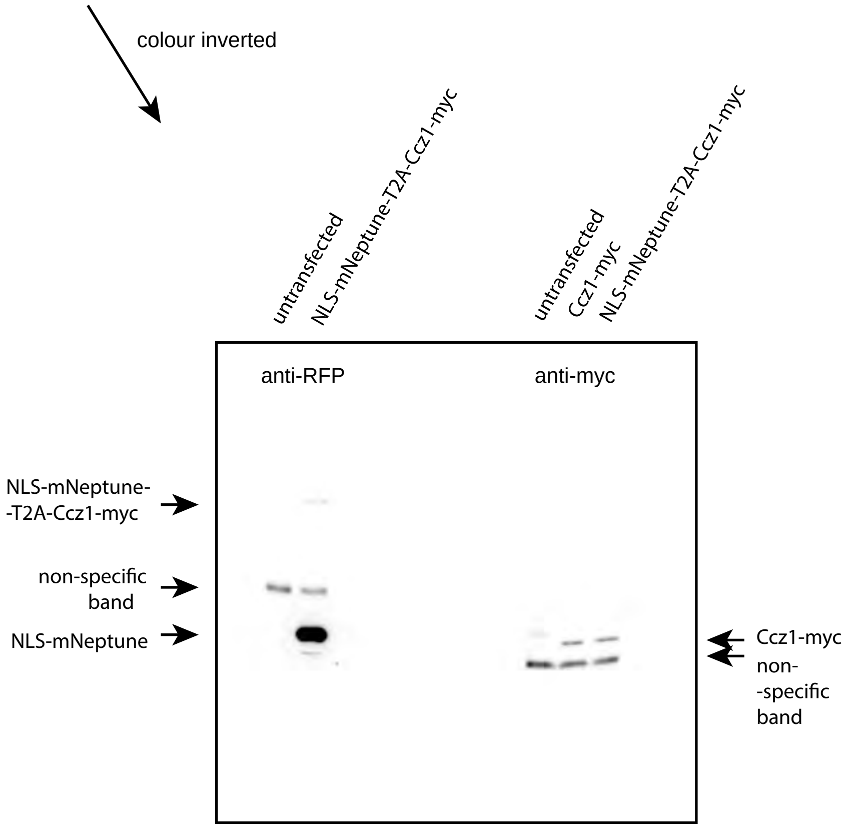

Membranes were first stained with anti-RFP / anti-myc.

ladder | exposure 1 s | exposure 60 s

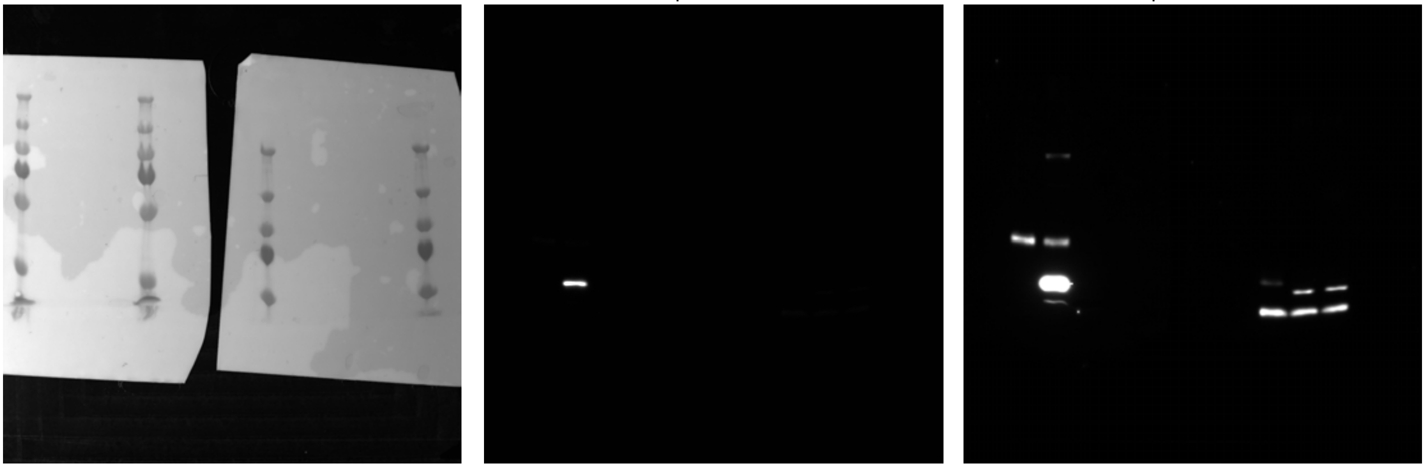

anti-RFP | anti-myc | anti-RFP | anti-myc

Membranes were subsequently stained with anti-actin without stripping.
Actin staining is a lot stronger, so the additional band appears at short exposure.

ladder | exposure 1 s | exposure 60 s

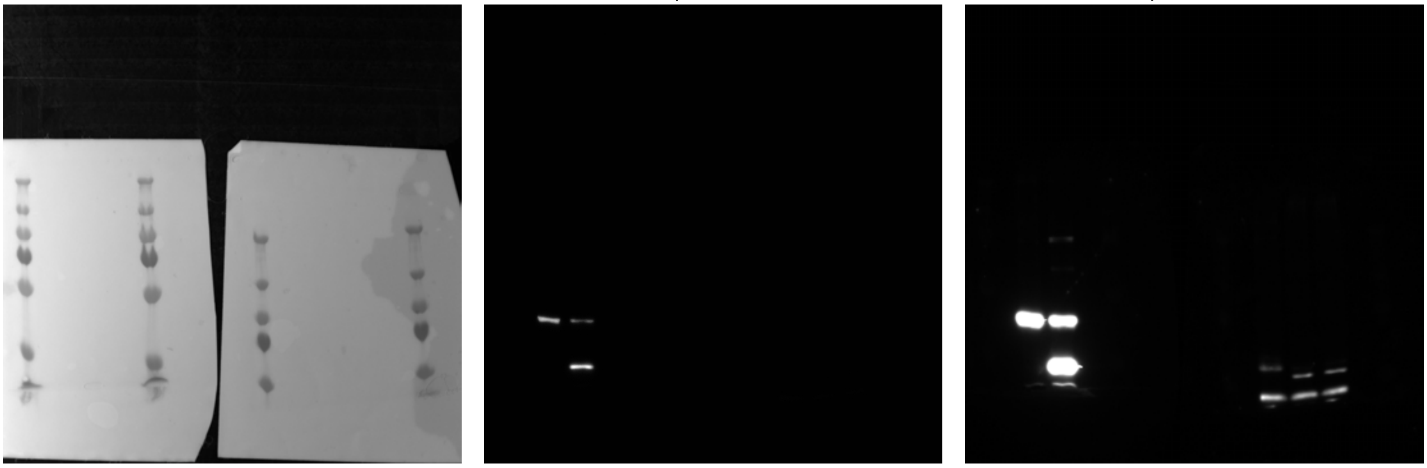

10% gel | 7.5% gel | anti-actin | anti-actin | anti-actin | anti-actin

ThermoFisher PageRuler Plus
pre-stained protein ladder

colour inverted

colour inverted

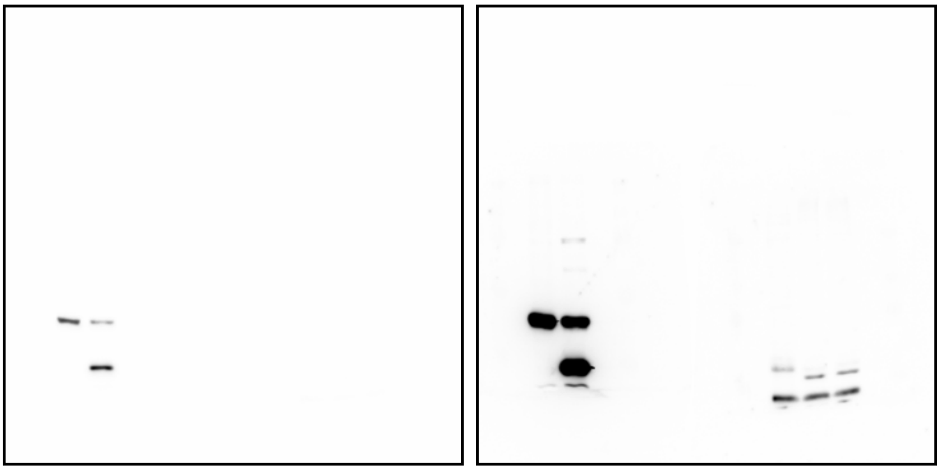

Actin partially ran off the gel, but the
non-specific band shows equal loading.
