# Supplementary material for: A novel live-cell imaging assay reveals regulation of endosome maturation
Source: eLife. 2021 Nov 30;10:e70982. doi: 10.7554/eLife.70982 (PMC8635980; doi:10.7554/eLife.70982)
Supplement: Supplementary file 1. — Table to specify oligonucleotide sequences and their description and purpose in generating constructs as outlined in Materials and methods. [file elife-70982-supp1.docx]

**Table S1. Oligonucleotide sequences used to generate specified constructs**

| **Oligonucleotide description/ purpose** | **Oligonucleotide sequence(s)** | |
| --- | --- | --- |
| **Generate ccz1 plasmid without myc tag using site-directed mutagenesis** | | |
| Remove myc from ccz1-myc plasmid | taaacggccggccgcggt | atccaagaagaagatgttgttgaactgcgttgcac |
| **Generate mNeptune2 plasmid with NLS inserted at N-terminal using site-directed mutagenesis** | | |
| Append NLS to mNeptune2 | gcggaaggtcgtgtctaagggcgaagagc | ttcttctttggcatggtggcgaccggtag |
| **Generate NLS-mNeptune2-5-T2A-ccz1 plasmid using NEBuilder HiFi Assembly** | | |
| Amplify NLS-mNeptune2 | tgccgaattccttgtacagctcgtccatg | gatctgccgccgcgatcgccatgccaaagaagaagcgg |
| Amplify T2A from px458 | ccggccgccgctgcagccattgggccaggattctcctc | gctgtacaaggaattcggcagtggagag |
| Amplify ccz1 plasmid | ggcgatcgcggcggcagatc | atggctgcagcggcggcc |
| **Generate Topo plasmids with mTurquoise2 and EYFP sequences** | | |
| mTurquoise2 +half-the-linker sequence | atggtgagcaagggcgaggagctgttcaccggggtggtgcccatcctggtcgagctggacggcgacgtaaacggccacaagttcagcgtgtccggcgagggcgagggcgatgccacctacggcaagctgaccctgaagttcatctgcaccaccggcaagctgcccgtgccctggcccaccctcgtgaccaccctgtcctggggcgtgcagtgcttcgcccgctaccccgaccacatgaagcagcacgacttcttcaagtccgccatgcccgaaggctacgtccaggagcgcaccatcttcttcaaggacgacggcaactacaagacccgcgccgaggtgaagttcgagggcgacaccctggtgaaccgcatcgagctgaagggcatcgacttcaaggaggacggcaacatcctggggcacaagctggagtacaactacttcagcgacaacgtctatatcaccgccgacaagcagaagaacggcatcaaggccaacttcaagatccgccacaacatcgaggacggcggcgtgcagctcgccgaccactaccagcagaacacccccatcggcgacggccccgtgctgctgcccgacaaccactacctgagcacccagtccaagctgagcaaagaccccaacgagaagcgcgatcacatggtcctgctggagttcgtgaccgccgccgggatcactctcggcatggacgagctgtacaagGGTGGAGGCG | |
| half-the-linker +EYFP sequence | GTAGCGAATTCatggtgagcaagggcgaggagctgttcaccggggtggtgcccatcctggtcgagctggacggcgacgtaaacggccacaagttcagcgtgtccggcgagggcgagggcgatgccacctacggcaagctgaccctgaagttcatctgcaccaccggcaagctgcccgtgccctggcccaccctcgtgaccaccttcggctacggcctgcagtgcttcgcccgctaccccgaccacatgaagcagcacgacttcttcaagtccgccatgcccgaaggctacgtccaggagcgcaccatcttcttcaaggacgacggcaactacaagacccgcgccgaggtgaagttcgagggcgacaccctggtgaaccgcatcgagctgaagggcatcgacttcaaggaggacggcaacatcctggggcacaagctggagtacaactacaacagccacaacgtctatatcatggccgacaagcagaagaacggcatcaaggtgaacttcaagatccgccacaacatcgaggacggcagcgtgcagctcgccgaccactaccagcagaacacccccatcggcgacggccccgtgctgctgcccgacaaccactacctgagctaccagtccgccctgagcaaagaccccaacgagaagcgcgatcacatggtcctgctggagttcgtgaccgccgccgggatcactctcggcatggacgagctgtacaagtaa | |
| **Generate GalT-pHlemon plasmid using NEBuilder HiFi Assembly (substitute mCherry with pHlemon)** | | |
| Amplify GalT plasmid minus mCherry | agcggccgcgactctagat | ggtggcgaccggtggatc |
| Amplify mTurquoise2 | aggatccaccggtcgccaccatggtgagcaagggcgag | aattcgctaccgcctccacccttgtacag |
| Amplify EYFP | ggtggaggcggtagcgaattcatggtgag | gatctagagtcgcggccgctttacttgtacagctcgtc |
| **Generate GalT-EGFP plasmid using NEBuilder HiFi Assembly (substitute mCherry with EGFP)** | | |
| Amplify GalT plasmid minus mCherry | agcggccgcgactctagat | ggtggcgaccggtggatc |
| Amplify EGFP from Lamp1-EGFP | aggatccaccggtcgccaccatggtgagcaagggcgag | gatctagagtcgcggccgctttacttgtacagctcgtccatg |
