# Supplementary material for: A novel live-cell imaging assay reveals regulation of endosome maturation
Source: eLife. 2021 Nov 30;10:e70982. doi: 10.7554/eLife.70982 (PMC8635980; doi:10.7554/eLife.70982)
Supplement: Transparent reporting form [file elife-70982-transrepform1.docx]

***eLife’s* transparent reporting form**

We encourage authors to provide detailed information *within their submission* to facilitate the interpretation and replication of experiments. Authors can upload supporting documentation to indicate the use of appropriate reporting guidelines for health-related research (see [EQUATOR Network](http://www.equator-network.org/%20)), life science research (see the [BioSharing Information Resource](https://biosharing.org/)), or the [ARRIVE guidelines](http://www.plosbiology.org/article/info:doi/10.1371/journal.pbio.1000412) for reporting work involving animal research. Where applicable, authors should refer to any relevant reporting standards documents in this form.

If you have any questions, please consult our Journal Policies and/or contact us: [editorial@elifesciences.org](mailto:editorial@elifesciences.org).

**Sample-size estimation**

- You should state whether an appropriate sample size was computed when the study was being designed
- You should state the statistical method of sample size computation and any required assumptions
- If no explicit power analysis was used, you should describe how you decided what sample (replicate) size (number) to use

Please outline where this information can be found within the submission (e.g., sections or figure legends), or explain why this information doesn’t apply to your submission:

No explicit power analysis was used. After a pilot quantification, it was deemed sufficient to analyze 10-20 endosomes per experiment – any higher numbers improved the noise of the averaged data but did not change the results of the quantification. The endosomes were selected from at least 3-10 different cells per experiment to ensure reproducibility between cells. This information can be found in individual figure legends and in methods section “single endosome analysis and quantification” subsection.

**Replicates**

- You should report how often each experiment was performed
- You should include a definition of biological versus technical replication
- The data obtained should be provided and sufficient information should be provided to indicate the number of independent biological and/or technical replicates
- If you encountered any outliers, you should describe how these were handled
- Criteria for exclusion/inclusion of data should be clearly stated
- High-throughput sequence data should be uploaded before submission, with a private link for reviewers provided (these are available from both GEO and ArrayExpress)

Please outline where this information can be found within the submission (e.g., sections or figure legends), or explain why this information doesn’t apply to your submission:

Each experiment was repeated three times (three independent biological replicates), and given the reproducibility of results, did not need to be repeated further. The biological replicates were performed on different days using different clones (ccz1 WT/KO) or independent transiently transfected cells. This information can be found in individual figure legends and in methods section “single endosome analysis and quantification” subsection. Criteria for inclusion/exclusion of endosomes from analysis is also included in the “single endosome analysis and quantification” subsection.

**Statistical reporting**

- Statistical analysis methods should be described and justified
- Raw data should be presented in figures whenever informative to do so (typically when N per group is less than 10)
- For each experiment, you should identify the statistical tests used, exact values of N, definitions of center, methods of multiple test correction, and dispersion and precision measures (e.g., mean, median, SD, SEM, confidence intervals; and, for the major substantive results, a measure of effect size (e.g., Pearson's r, Cohen's d)
- Report exact p-values wherever possible alongside the summary statistics and 95% confidence intervals. These should be reported for all key questions and not only when the p-value is less than 0.05.

Please outline where this information can be found within the submission (e.g., sections or figure legends), or explain why this information doesn’t apply to your submission:

Where appropriate, means, standard deviations, SEM, Pearson’s correlation and linear regression were calculated using GraphPad Prism. This information, together with sample size, can be found in individual figure legends and in methods section “single endosome analysis and quantification” subsection.

(For large datasets, or papers with a very large number of statistical tests, you may upload a single table file with tests, Ns, etc., with reference to sections in the manuscript.)

**Group allocation**

- Indicate how samples were allocated into experimental groups (in the case of clinical studies, please specify allocation to treatment method); if randomization was used, please also state if restricted randomization was applied
- Indicate if masking was used during group allocation, data collection and/or data analysis

Please outline where this information can be found within the submission (e.g., sections or figure legends), or explain why this information doesn’t apply to your submission:

The only group allocation was performed on quantified endosomes in GFP-Snx1+mApple-Rab7 expressing cells, to bin them into three patterns of a single peak, double peak or continuous peak. This was done by eye. The description of categories is included in the results section “Snx1 recruitment is initiated with Rab5 recruitment and can persist during Rab7 stages” subsection and in the corresponding figure legend.

**Additional data files (“source data”)**

- We encourage you to upload relevant additional data files, such as numerical data that are represented as a graph in a figure, or as a summary table
- Where provided, these should be in the most useful format, and they can be uploaded as “Source data” files linked to a main figure or table
- Include model definition files including the full list of parameters used
- Include code used for data analysis (e.g., R, MatLab)
- Avoid stating that data files are “available upon request”

Please indicate the figures or tables for which source data files have been provided:

Figure 2, Figure Supplement 1C – Source Data 1. Cell growth numbers and quantification post nigericin treatment.

Figure 4D - Source Data 1. Quantification of Rab5 and Rab7 recruitment at endosomes.

Figure 5C - Source Data 1. Quantification of Rab5 and PI(3)P recruitment at endosomes.

Figure 6C - Source Data 1. Quantification of Rab5 and Snx1 recruitment at endosomes.

Figure 6E - Source Data 1. Quantification of Rab5 and Snx1 subdomains at endosomes.

Figure 6, Figure supplement 1C - Source Data 1. Quantification of Rab5 and Snx1 subdomains at endosomes.

Figure 6, Figure supplement 1E - Source Data 1. Quantification of Rab7 and Snx1 subdomains at endosomes.

Figure 7 - Source Data 1. Quantification of Rab7 and Snx1 recruitment at endosomes.

Figure 8C - Source Data 1. Quantification of Rab5 and Rab11 recruitment at endosomes.

Figure 8D - Source Data 1. Quantification of Rab5 and Rab11 subdomains at endosomes.

Figure 8G - Source Data 1. Quantification of Rab7 and Rab11 recruitment at endosomes.

Figure 8H - Source data 1. Quantification of Rab7 and Rab11 subdomains at endosomes.

Figure 8, Figure supplement 1A and C – Source data 1. Numerical data for all analysed endosomes plotted in Figure 8, Figure supplement 1B.

Figure 9G – Source Data 1. Quantification of acquisition and removal of TfR to and from endosomes in relation to Rab5.

Figure 9H – Source Data 1. Quantification of acquisition and removal of CDMPR to and from endosomes in relation to Rab5.

Figure 9G – Source Data 1. Quantification of acquisition and removal of GalT to and from endosomes in relation to Rab5.

Figure 10D - Source Data 1. Quantification of GalT-pHlemon signal in cells in calibration buffers of known pH.

Figure 10E - Source Data 1. Quantification of GalT-pHlemon signal in Golgi ribbon, endo-/lysosomal puncta, and enlarged endosomes.

Figure 10, Figure supplement 1A - Source Data 1. Sigmoidal dose-response curve to define relationship between GalT-pHlemon signal and pH.

Figure 11C - Source Data 1. Quantification of Rab5 recruitment and GalT-pHlemon signal at endosomes.

Figure 11F - Source data 1. Quantification of Rab7 recruitment and GalT-pHlemon signal at endosomes.

Figure 12 - Source Data 1. Quantification of Rab5 recruitment and GalT-pHlemon signal at endosomes in Ccz1 WT, KO and rescue cells.

Figure 12, Figure supplement 1A - Source Data 1. Raw RT-PCR data for Ccz1 expression levels in Ccz1 WT vs KO cells.

Figure12, Figure supplement 2A - Source Data 1. Original Western Blot images for NLS-mNeptune-T2A-myc expression.

Figure 13B - Source Data 1. Quantification of GalT-pHlemon signal in endo-/lysosomal puncta in Ccz1 WT, KO and rescue cells.

Figure 13D - Source Data 1. Quantification of GalT-pHlemon signal in endo-/lysosomal puncta in Ccz1 WT, KO and rescue cells post nigericin treatment.
