# Supplementary material for: A novel live-cell imaging assay reveals regulation of endosome maturation
Source: eLife. 2021 Nov 30;10:e70982. doi: 10.7554/eLife.70982 (PMC8635980; doi:10.7554/eLife.70982)
Supplement: Peer review history

# Editor report: Editor's evaluation

**DOI:** 10.7554/eLife.70982.sa0

**Authors:** Christopher G Burd

**Affiliations:** Yale School of Medicine United States

The manuscript describes and validates a method for observing endosome dynamics in living cells. The approach may open the door to mechanistic studies of endosome maturation.

# Decision letter

**DOI:** 10.7554/eLife.70982.sa1

**Affiliations:** Yale School of Medicine United States; Yale School of Medicine United States; The University of Chicago United States

Our editorial process produces two outputs: (i) public reviews designed to be posted alongside the preprint for the benefit of readers; (ii) feedback on the manuscript for the authors, including requests for revisions, shown below. We also include an acceptance summary that explains what the editors found interesting or important about the work.

**Decision letter after peer review:**

Thank you for submitting your article "A novel live cell imaging assay reveals regulation of endosome maturation" for consideration by *eLife*. Your article has been reviewed by 3 peer reviewers, including Christopher G Burd as Reviewing Editor and Reviewer #1, and the evaluation has been overseen by Vivek Malhotra as the Senior Editor. The following individual involved in review of your submission has agreed to reveal their identity: Benjamin S Glick (Reviewer #3).

The reviewers have discussed their reviews with one another, and the Reviewing Editor has drafted this to help you prepare a revised submission.

Essential revisions:

The authors describe a technical innovation that swells endosomes and the terminal compartments of the Golgi of cultured human cells, allowing them to observe the maturation of the dramatically enlarged compartments by fluorescence microscopy. Ionophore treatment is a significant perturbation, but a variety of findings validate that the enlarged endosomes undergo Rab5-to-Rab7 conversion over a time course that is roughly similar to the rate of Rab endosome conversion in unperturbed cells. The results further establish that endosome recruitment of a recycling/retrograde cargo sorting factor, Snx1, is not correlated with the Rab5-to-Rab7 early-to-late conversion process. Endosomes decorated with another regulator of recycling, Rab11, are reported to make transient contact with Rab-negative and Rab5/7-positive endosomes, leading the authors conclude that cargo recycling/retrograde sorting pathways are unaffected by nigericin-induced swelling. In contrast, acidification of the endosome is reported to be linked to Rab5-to-Rab7 conversion. It is further reported that the terminal compartments of the Golgi apparatus, identified by the presence of GalT, are swelled by nigericin and they are observed to fuse with a Rab5-negative endosome which subsequently acquires Rab5, initiating to Rab5-to-Rab7 conversion. Overall, this is a high-quality study that presents a useful tool for observing endosome dynamics in live cells. The approach was used to elucidate several unappreciated aspects of endosome maturation and Golgi-endosome interactions that are intriguing though largely preliminary in significance.

1. A critical concern throughout the manuscript regards extrapolations of endosome protein recruitment as a direct indication of cargo transport to or out of an endosome. No cargo transport assays are included in the study, and the reviewers concluded that important points, described below, are not firmly substantiated.

A) It is asserted that the Rab conversion process under investigation indicates physiological trafficking of endocytosed cargo to the lysosome via endosome maturation. However, cargo trafficking and/or turnover was not examined in the study. One dextran uptake experiment suggested that some dextran-containing compartments become decorated with tagged Rab5, but the prevalence of this could not be ascertained from the presentation and transport to the lysosome was not tested. This could be accomplished by examining endocytosis and subsequent trafficking using a fluorescent cargo (e.g., dextran, LDL).

B) It is asserted that recruitment of tagged Snx1 or Rab11 to the endosome indicates recycling of endocytosed cargo to the plasma membrane. However, trafficking of a recycling cargo protein was not examined. Testing if recycling cargo is exported from the endosome via Snx1-coated tubules or under Rab11 regulation is necessary to support the conclusions regarding export of cargo from the endosome. This is especially important because many of the reporter proteins are tagged with a fluorescent protein and expressed transiently at high level in cells that also express the native proteins. It is also important because presumed changes in the tension of the plasma membrane of rapidly swelled endosomes may affect the formation of carriers of recycling pathways, such as documented for phagosomes during their maturation (Freeman et al., 2020).

It is noted that accurate descriptions of findings are included in the text (e.g., lines 71-72 summarize the findings as "recruitment" rather than cargo trafficking/recycling to the PM), however, equating endosome recruitment of sorting factors with cargo trafficking is the norm throughout the manuscript, including in the Abstract.

2. The conclusion that Rab11+ compartments directly contact Rab5-negative and Rab5/7-positive endosomes is not convincingly supported by the data. The Rab11 fluorescence signals are very complex, with so many puncta present throughout the crowded cytoplasm that specific (i.e., not incidental) contacts between Rab11-decorated endosomes and early endosomes were not (perhaps cannot) distinguished by the approach used. The observations may also be influenced by the expression level of GFP-Rab11. The reviewers considered these conclusions to be preliminary in significance.

3. The conclusion that entire late Golgi compartments are directed into the endosome maturation pathway is poorly supported by the data. This aspect of the study is provocative, yet the reviewers consider this aspect of the study to be preliminary in significance. This finding was not even addressed in the Discussion.

(A) The critical observations are determinations of the localization of GalT-fluorescent fusion proteins in nigericin-treated cells. Residents of trans/TGN Golgi compartments cycle between the Golgi and the endosome. Golgi residents that fail to be sorted into the retrograde pathway continue to the lysosome and are degraded (via the MVB pathway). While the authors' observations might indicate the convergence of the secretory and endosome maturation pathways, other possibilities were not addressed. It may be that the retrograde pathway fails to some degree when late Golgi compartments are swollen (see also point 1), resulting in 'leakage' of GalT fusion proteins to the lysosome. This is especially concerning because most of the experiments employed GalT fusion proteins where the increased expression may saturate retrograde pathways.

(B) The time required acquisition of Rab5 on GalT-positive compartments is long – on the order of 1-2 hours. It is unclear if the observations of swollen late Golgi compartments reveal a physiologically meaningful transition.

4. The conclusion that endosome acidification is linked to Rab5-to-Rab7 conversion is not convincingly supported by the data. The graphical presentations of the data shown in figures 10 C, E, F indicate that acidification was initiated on Rab5-positive endosomes and that the rates of acidification did not change once Rab7 was acquired. On the other hand, the data presented in figures 10B and 11B are suggestive of a temporal correlation between acidification and the acquisition of Rab7. This discrepancy needs to be resolved.

*Reviewer #1 (Recommendations for the authors):*

I have a few concerns regarding some other points made using the approach.

1. The analyses of rab11, Snx1, and endosome acidification are interesting, though the accuracy of such analyses is not rigorously validated at this stage due to the small size of native endosomes. Hence, it is unclear if the conclusions apply to native endosomes. Related, a recent paper from Sergio Grinstein and colleagues (Freeman et al., 2020) showed that ion transport-driven changes in membrane tension during phagosome maturation (membrane "crenation") are necessary to trigger Snx1, Snx2 and Snx5 recruitment to the phagosome. This may not apply to native endosomes, but the physical principal (ie, 'crenation') is applicable to any membrane compartment, so there is good reason to be concerned about confounding effects due to the swelling of these compartments.

2. I am not convinced that it has been shown that swelled trans/TGN compartments provides the membrane of rab5 and rab7 positive endosomes. Figure 2 shows that a compartment, probably the TGN, is swelled by nigericin and it is argued (lines ~155-162) that the presence of overexpressed (and tagged) GalT-GFP in ILVs of MVBs probably indicates that rab5 and rab7 positive endosomes are derived from the swollen trans Golgi/TGN. Proteins considered to be residents of the trans/TGN compartments cycle between the endosome and the Golgi and Golgi residents that fail to be sorted into the retrograde pathway continue to the lysosome and are degraded (via the MVB pathway). It may be that the retrograde pathway fails to some degree when all of these compartments are swollen (point 1). In my opinion, the paper would be improved by removing this material.

*Reviewer #2 (Recommendations for the authors):*

(1) The most serious problem is with the data and conclusions related to Rab11 interaction with early endosomes in Figure 8. Unlike the Snx1 data, where association with the early endosomes is fairly convincing, there are simply too many Rab11 puncta in the cell to tell if they are meaningfully interacting with the early endosomes, or if the data simply reflects random collisions of small recycling endosomes with the enlarged early endosomes. To determine if these collisions are functionally significant the authors would need to show data on the transfer of recycling cargo from Rab5 endosomes to Rab11 endosomes during such interactions. Without this I would recommend removing this data from the paper. Along these lines, while the Snx1 data was more convincing, it was overinterpreted as "recycling" or "sorting" even though no cargo was followed. The authors should more carefully word the text, especially the abstract and discussion, to avoid such gross overinterpretation of the Snx1 data.

(2) To really establish this endosome maturation model the authors should establish if the enlarged endosomes contain endocytosed cargo, as opposed to Golgi-derived cargo, and determine how long it takes to acquire such cargo. This could be accomplished using Tf, EGF, or perhaps dextran at early timepoints after nigericin washout.

(3) Figure 7 – It was not convincing that data in panels F and G are different from each other.

(4) Figure 11 – I find the interpretation of panel D confusing. How can we interpret this as connected to Rab conversion when even the labeled compartments at the earliest time point in the czz1 knockout have abnormally high pH, and during the time-course even the last timepoint for czz1 KO is higher than that of the earliest timepoint for WT?

(5) Figure 12 – The criteria used to determine which GalT structures are Golgi or lysosomes seems flawed. Morphology alone is not sufficient to identify the compartments with high accuracy, especially after perturbation. Also, to what extent does GalT-CFP label lysosomes without nigericin treatment?

*Reviewer #3 (Recommendations for the authors):*

– What is the status of recycling endosomes? These structures must have some relationship to maturing endosomes but the literature is confusing. Rab11 is the traditional marker for recycling endosomes, and the authors describe Rab11 vesicles that "contact" maturing endosomes. How is this pathway envisioned to occur? Does internalized material ever accumulate in Rab11-positive recycling endosomes? It would be useful to bring the earlier work on the FERARI complex into a model that incorporates the current data.

– Do late endosomes fuse completely with lysosomes, or do they typically undergo kiss-and-run fusion as has been described in both mammalian and yeast cells? Some of the images here seem to argue against complete fusion. What are the implications? Specifically, if late endosomes are constantly being generated by maturation but are not consumed by fusion with lysosomes, why doesn't the cell fill up with late endosomes?

– The most striking result is that TGN-derived compartments go on to become Rab5-labeled endosomes, yet this topic is not even mentioned in the Discussion. Do the authors infer that there is a close relationship between the TGN and early endosomes, as has been observed in plant and yeast cells? Or is it more likely that existing Rab5-positive early endosomes become more prominent due to fusion with enlarged TGN-derived compartments?

In Figure 1D, homotypic fusion is readily apparent but I don't see the Rab conversion described in the legend.

The videos are hard to match to the figures. For example, the "Figure 1C supplement 1" video seems to have nothing to do with Figure 1C, and I couldn't find a description of this video.

Figure 5: PI(3)P is presumably generated, not recruited.

# Reply: Author response

**DOI:** 10.7554/eLife.70982.sa2

Essential revisions:

The authors describe a technical innovation that swells endosomes and the terminal compartments of the Golgi of cultured human cells, allowing them to observe the maturation of the dramatically enlarged compartments by fluorescence microscopy. Ionophore treatment is a significant perturbation, but a variety of findings validate that the enlarged endosomes undergo Rab5-to-Rab7 conversion over a time course that is roughly similar to the rate of Rab endosome conversion in unperturbed cells. The results further establish that endosome recruitment of a recycling/retrograde cargo sorting factor, Snx1, is not correlated with the Rab5-to-Rab7 early-to-late conversion process. Endosomes decorated with another regulator of recycling, Rab11, are reported to make transient contact with Rab-negative and Rab5/7-positive endosomes, leading the authors conclude that cargo recycling/retrograde sorting pathways are unaffected by nigericin-induced swelling. In contrast, acidification of the endosome is reported to be linked to Rab5-to-Rab7 conversion. It is further reported that the terminal compartments of the Golgi apparatus, identified by the presence of GalT, are swelled by nigericin and they are observed to fuse with a Rab5-negative endosome which subsequently acquires Rab5, initiating to Rab5-to-Rab7 conversion. Overall, this is a high-quality study that presents a useful tool for observing endosome dynamics in live cells. The approach was used to elucidate several unappreciated aspects of endosome maturation and Golgi-endosome interactions that are intriguing though largely preliminary in significance.

1. A critical concern throughout the manuscript regards extrapolations of endosome protein recruitment as a direct indication of cargo transport to or out of an endosome. No cargo transport assays are included in the study, and the reviewers concluded that important points, described below, are not firmly substantiated.

A) It is asserted that the Rab conversion process under investigation indicates physiological trafficking of endocytosed cargo to the lysosome via endosome maturation. However, cargo trafficking and/or turnover was not examined in the study. One dextran uptake experiment suggested that some dextran-containing compartments become decorated with tagged Rab5, but the prevalence of this could not be ascertained from the presentation and transport to the lysosome was not tested. This could be accomplished by examining endocytosis and subsequent trafficking using a fluorescent cargo (e.g., dextran, LDL).

To further examine cargo trafficking, we performed additional dextran-Alexa647 uptake assays in mApple-Rab5 and GFP-Rab7 expressing cells. The experiments revealed that dextran-Alexa647 is present in Rab5-positive endocytic vesicles that go on to fuse with Rab5-negative enlarged structures, which subsequently become Rab5 positive. Those Rab5 enlarged endosomes undergo Rab conversion, turn Rab7 positive and form endolysosomes, which mature to lysosomes, still containing the dextran. These data are now included, replacing the shorter dextran-Alexa488/mApple-Rab5 panel (Figure 3, figure supplement 1B).

As for the prevalence of endocytic Dextran-containing compartments, we now include a higher-quality image set, showing Rab5-positive enlarged endosomes, both with and without detectable Dextran-AF488, as well as Dextran-containing Rab5-negative (presumably Rab-converted) endosomes. We cannot really measure prevalence because Dextran endocytosis is too fast to capture its early and highly-synchronized fusion events with the asynchronously-generated and highly-transient Rab5-positive enlarged compartments. (Figure 3, figure supplement 1A).

As a second piece of evidence, we transfected cells with BFP-Rab5 and transferrin receptor tagged to GFP (TfR-GFP). We then added a GFP-nanobody, which was tagged with mCherry, resulting in illumination of surface TfR and its subsequent trafficking. Already within a few minutes after addition of the nanobody, we were able to detect the nanobody together with Rab5 in close proximity of the enlarged endosomes; the presence of the nanobody on the limiting membrane became more obvious after about 15-20 min of the addition. Thus, plasma membrane derived cargo appears to reach the enlarged endosome in a reasonable time frame. Proper quantification over many endosomes as we did for the other experiments proved to be difficult because BFP-Rab5 seems to be less well tolerated by cells compared to the GFP or mApple versions, and only a few cells with a weakly detectable Rab5 expression could be imaged. Further difficulties stem from the short window of opportunity to trace the synchronous uptake of nanobody-tagged surface TfR and its trafficking to the highly-asynchronous and transient Rab5-positive enlarged endosomes. Moreover, the signal for the mCherry-tagged GFP-nanobody was rather weak, limiting the number of endosomes with detectable TfR. Nevertheless, we observed multiple events and two examples are now shown in the manuscript in supplementary information (Figure 3, figure supplement 2). Additionally, we show quantification for TfR-GFP acquisition to and removal from the enlarged endosomes relative to Rab5 recruitment (Figure 9A,D,G). These data show concomitant arrival of Rab5 and TfR to the endosome and prompt removal of TfR as the endosome undergoes Rab conversion.

Taken together, we provide more evidence that cargo from the plasma membrane reaches the enlarged early endosomal structures and that we can recapitulate the lifetime of an endosome from early-to-late to endolysosome to lysosome.

B) It is asserted that recruitment of tagged Snx1 or Rab11 to the endosome indicates recycling of endocytosed cargo to the plasma membrane. However, trafficking of a recycling cargo protein was not examined. Testing if recycling cargo is exported from the endosome via Snx1-coated tubules or under Rab11 regulation is necessary to support the conclusions regarding export of cargo from the endosome. This is especially important because many of the reporter proteins are tagged with a fluorescent protein and expressed transiently at high level in cells that also express the native proteins. It is also important because presumed changes in the tension of the plasma membrane of rapidly swelled endosomes may affect the formation of carriers of recycling pathways, such as documented for phagosomes during their maturation (Freeman et al., 2020).

We agree with the reviewers that we did not show any recycling cargo in the original manuscript. We now used TfR-GFP and mApple-Rab5 to identify the enlarged endosomes, from which TfR would recycle to the plasma membrane. We now include data in the manuscript showing that TfR-GFP is present in enlarged Rab5 positive endosomes and that the TfR-GFP signal gets incorporated into vesicles and goes down over time in the enlarged endosomes, consistent with recycling (Figure 9 A,D,G). Likewise, we used CDMPR-GFP, which recycles to the Golgi and we observed again a reduction of the CDMPR signal in Rab5 positive enlarged endosomes over time and the formation of CDMPR positive vesicles (Figure 9 B,E,H). Importantly, we did not observe the same reduction of the signal (or its packaging into vesicles) for GalT-GFP, which go down the route to the lysosome (Figure 9 C,F,I). Therefore, the reduction in the GFP signal cannot be explained by bleaching and thus we assume that we observe recycling of TfR and CDMPR to the plasma membrane and the Golgi, respectively. Regardless of the recycling pathway or the destination of the recycled cargo, these data indicate that sorting and recycling appear to be functional in the enlarged endosomes. We have not investigated whether TfR or CDMPR is exported away from the endosome via Snx1-coated tubules or Rab11-mediated transport carriers, as this would require triple transfection with BFP-Rab5, which cells do not tolerate well.

To provide evidence for the role of Snx1 or Rab11 in mediating recycling in the enlarged endosomes, we used CRISPR-Cas9 SNX1 and RAB11a KO cell lines. We did not see a strong effect on recycling of TfR-GFP, which might be due to redundancy issues. SNX4 can compensate for SNX1 and presumably Rab11b for Rab11a, and over time the cell lines potentially adapt to the loss of SNX1 and RAB11a, respectively. To have a more acute readout, we knocked down Rab11a and b with siRNAs. Unfortunately, we obtained mixed results as we observed a delay in TfR recycling upon Rab11a+b knockdown in some experiments but not in others. Of course, this might be related to the knockdown efficiency in individual experiments and in the experiments where we observed an effect the efficiency might have been higher. Even though we sometimes observed an effect, we do not feel comfortable putting these data into the manuscript. Since the direct correlation between Rab11/Snx1 and cargo recycling has not been shown, we therefore adjusted the manuscript text accordingly.

As with regards to potential changes in membrane tension and its effect on endosome recycling function, while we cannot exclude that these changes may occur because we did not measure them in our system, we now include data in the manuscript showing that uptake from the plasma membrane works well and that cargo is recycled away from the endosome (TfR and CDMPR data). The crenation, as described in Freeman et al., paper, is driven by changes in osmolarity, which the cells could promptly (within minutes) readjust after experimental intervention. As we allow recovery after the acute nigericin treatment, we predict there to be no big perturbations in osmolarity over the subsequent hours during which we can image cells and observe traffic events. Our assumptions here are supported by the stable endosome size during endosome maturation and the new evidence we provide on the cargo recycling.

It is noted that accurate descriptions of findings are included in the text (e.g., lines 71-72 summarize the findings as "recruitment" rather than cargo trafficking/recycling to the PM), however, equating endosome recruitment of sorting factors with cargo trafficking is the norm throughout the manuscript, including in the Abstract.

We assume that the reviewers mean lines 78-79. We changed the text to reflect that we determine the interaction of Rab11 vesicles with enlarged endosomes as recruitment. Even though we do not mention recycling to the plasma membrane explicitly in the abstract, we changed the wording in the abstract and are more careful throughout the manuscript not to mention any specific cargo recycling, unless warranted.

2. The conclusion that Rab11+ compartments directly contact Rab5-negative and Rab5/7-positive endosomes is not convincingly supported by the data. The Rab11 fluorescence signals are very complex, with so many puncta present throughout the crowded cytoplasm that specific (i.e., not incidental) contacts between Rab11-decorated endosomes and early endosomes were not (perhaps cannot) distinguished by the approach used. The observations may also be influenced by the expression level of GFP-Rab11. The reviewers considered these conclusions to be preliminary in significance.

To address the reviewers’ concern, we took videos in which images were taken in much shorter, 2-sec intervals. These videos show that Rab11 contact directly enlarged Rab5 negative and positive compartments and stay on there for various times, indicating that this interaction is not incidental. These data are now included in the manuscript. (video Figure 8A supplement 2 and 3).

3. The conclusion that entire late Golgi compartments are directed into the endosome maturation pathway is poorly supported by the data. This aspect of the study is provocative, yet the reviewers consider this aspect of the study to be preliminary in significance. This finding was not even addressed in the Discussion.

We apologize for the confusion. We agree with the reviewers that we cannot conclude that the entire late Golgi compartments are directed into the endosome maturation pathway as we can only have a qualitative but not quantitative measure. We changed the text to make this clearer and we now discuss the swelling of the TGN in the discussion. We are actually not the first ones to notice this. Already Tartak_off_ and Vassalli (J. Exp Med. 1977) reported the TGN swelling/dispersal after ionophore treatment, including nigericin. There were a few studies in the 1980’s and 1990’s (e.g. Vladutiu, BioSci. Report 1984, Merion and Sly, JCB 1983, which we cite in the manuscript), which report the swelling of the outer Golgi leaflet with additional cisternae getting affected with longer treatment, suggesting that our acute treatment would partially preserve the Golgi compartment. Indeed, this partial disruption of the Golgi agrees well with our data that recycling of CDMPR remains functional. Since the Golgi dispersal was well documented in the literature, we did not include this into the discussion. However, upon the reviewers’ suggestion, we now discuss our finding that Golgi compartments can fuel the endocytic pathway.

(A) The critical observations are determinations of the localization of GalT-fluorescent fusion proteins in nigericin-treated cells. Residents of trans/TGN Golgi compartments cycle between the Golgi and the endosome. Golgi residents that fail to be sorted into the retrograde pathway continue to the lysosome and are degraded (via the MVB pathway). While the authors' observations might indicate the convergence of the secretory and endosome maturation pathways, other possibilities were not addressed. It may be that the retrograde pathway fails to some degree when late Golgi compartments are swollen (see also point 1), resulting in 'leakage' of GalT fusion proteins to the lysosome. This is especially concerning because most of the experiments employed GalT fusion proteins where the increased expression may saturate retrograde pathways.

Indeed, we did not address the functionality of the retrograde transport to the TGN after nigericin washout in the original manuscript. The new experiments with the CDMPR described under point 1 would argue that the retrograde transport is functional at least at the level of cargo removal from the enlarged endosome. We can show that CDMPR positive structures bud off from the Rab5 positive enlarged endosomes and that the levels of CDMPR at the enlarged endosome drop over time. Moreover, these endosomes seem to be capable of also accepting CDMPR-containing vesicles. We take this as a strong indication that the Golgi-endosome shuttle is intact after the nigericin washout (Figure 9; Fig9, figure supplement 1).

If overexpression of proteins involved in the Golgi-endosome shuttle resulted in a saturated retrograde pathway, we would see a compromised removal of the overexpressed CDMPR-GFP from the endosome. However, this is not the case. In contrast to GalT constructs, all of the observed endosomes promptly removed CDMPR during or shortly before Rab5 recruitment. Thus, retention at the endosome and failure to recycle back to the Golgi is limited to the GalT fusion proteins.

Although endogenous GalT is rarely observed outside of the Golgi ribbon (Figure 3C), the constructs appear in punctate structures in addition to the Golgi ribbon, which occasionally colocalized with Rab5 and lysosomes (Figure 10B and C, Figure 3A). This suggests that GalT construct trafficking to the endosome is a property of the construct and not nigericin-mediated perturbation of retrograde trafficking. The combination of intact retrograde trafficking and the retention of GalT constructs in endosomes make GalT-pHlemon an excellent tool to study intralumenal pH of endosomes with minimum disruption to endosome function.

(B) The time required acquisition of Rab5 on GalT-positive compartments is long – on the order of 1-2 hours. It is unclear if the observations of swollen late Golgi compartments reveal a physiologically meaningful transition.

We can detect this also at earlier timepoints. We wanted the cells to reestablish the proper pH, and setting up the microscope (we record multiple cells simultaneously) takes some time. To be consistent, we usually start imaging 40 min after washout. We now include a time-lapse image series, in which Rab5 is recruited to an enlarged endosome, which then converts into a Rab7-positive endosome, within 20 min after nigericin washout (Figure 1, figure supplement 1A). Thus, these events happen already very early after nigericin washout and continue for hours. There is no synchronous wave after washout, rather we observe asynchronous events. Additionally, as the Golgi swell up, the early swollen compartments are easy to miss as they are frequently in different focal planes to smaller endosomes. As the cell adjusts to its new physiology, the enlarged compartments rearrange to sit in a single focal plane and are more practical to image en masse. We clarify this in the text.

4. The conclusion that endosome acidification is linked to Rab5-to-Rab7 conversion is not convincingly supported by the data. The graphical presentations of the data shown in figures 10 C, E, F indicate that acidification was initiated on Rab5-positive endosomes and that the rates of acidification did not change once Rab7 was acquired. On the other hand, the data presented in figures 10B and 11B are suggestive of a temporal correlation between acidification and the acquisition of Rab7. This discrepancy needs to be resolved.

We think that the issue might be related to the way the experiments were performed. In Figure 10 (now Figure 11), we used HeLa cells stably expressing either mApple-Rab5 or mApple-Rab7, while in Figure 11 (now Figure 12) Rab5 and Rab7 were transiently expressed in the ccz1 KO cell lines. To be consistent we repeated the experiments shown in Figure 10 (now Figure 11) with transient expression. We generated a new data set, which shows stabilization of pH upon Rab conversion, and now all data are consistent.

Reviewer #1 (Recommendations for the authors):

I have a few concerns regarding some other points made using the approach.

1. The analyses of rab11, Snx1, and endosome acidification are interesting, though the accuracy of such analyses is not rigorously validated at this stage due to the small size of native endosomes. Hence, it is unclear if the conclusions apply to native endosomes. Related, a recent paper from Sergio Grinstein and colleagues (Freeman et al., 2020) showed that ion transport-driven changes in membrane tension during phagosome maturation (membrane "crenation") are necessary to trigger Snx1, Snx2 and Snx5 recruitment to the phagosome. This may not apply to native endosomes, but the physical principal (ie, 'crenation') is applicable to any membrane compartment, so there is good reason to be concerned about confounding effects due to the swelling of these compartments.

Indeed, the Freeman et al., 2020 paper (as well as the Mercier, Lerios et al., 2020 NCB paper) explore the idea of osmotic/ionic pressure (and the associated membrane tension) changes playing a role in sorting and recycling, which could be of relevance to our system where the swollen Golgi likely arise from osmotic/ionic imbalances during nigericin treatment. The swelling in our system is most likely driven by interference to the proton gradient, since the protonophore FCCP inhibited monensin-induced swelling (Boss, Morre and Mollenhauer 1984 Eur J Cell Biol), and we observed a similar effect with V-ATPase inhibitor bafilomycin abolishing nigericin-induced swelling (data not shown). However, unlike the above-mentioned studies, where treatments were continuous, the nigericin treatment in our set up is highly transient and we allow our cells to recover and re-establish pH, thereby minimizing the possibility of any ionic imbalances during the observation phase. The macropinosomes in the Freeman et al., study respond to external ionic/osmotic stimuli within minutes of their addition, strongly suggesting that any ionic imbalances within our system would also be resolved within minutes of nigericin removal. Consistent with this notion, the enlarged endosomes do not rapidly shrink with the recruitment of Snx1, and rather remain stable in size until the endolysosomal stages. The mechanism driving the enlarged endosome shrinkage during endolysosomal stages will be subject of future studies. Although we do not show the functionality of Snx1 directly, we now include the CDMPR and TfR data to show that cargo recycling is functional at the enlarged endosomes. (Figure 9).

2. I am not convinced that it has been shown that swelled trans/TGN compartments provides the membrane of rab5 and rab7 positive endosomes. Figure 2 shows that a compartment, probably the TGN, is swelled by nigericin and it is argued (lines ~155-162) that the presence of overexpressed (and tagged) GalT-GFP in ILVs of MVBs probably indicates that rab5 and rab7 positive endosomes are derived from the swollen trans Golgi/TGN. Proteins considered to be residents of the trans/TGN compartments cycle between the endosome and the Golgi and Golgi residents that fail to be sorted into the retrograde pathway continue to the lysosome and are degraded (via the MVB pathway). It may be that the retrograde pathway fails to some degree when all of these compartments are swollen (point 1). In my opinion, the paper would be improved by removing this material.

Indeed, Figure 2 alone is not sufficient to argue that the enlarged endosomes derive from TGN-derived compartments. This is why we back up our hypothesis with Figure 3A, which shows a vesiculated GalT-GFP positive compartment acquire Rab5. To rule out any potential failure of retrograde transport, we now include CDMPR data, which shows prompt removal of CDMPR from the enlarged early endosome (Figure 9B,E,H). We do consider it important to leave these data in the manuscript because they provide also the basis for the use of GalT-pH lemon with which explore endosomal acidification.

Reviewer #2 (Recommendations for the authors):

(1) The most serious problem is with the data and conclusions related to Rab11 interaction with early endosomes in Figure 8. Unlike the Snx1 data, where association with the early endosomes is fairly convincing, there are simply too many Rab11 puncta in the cell to tell if they are meaningfully interacting with the early endosomes, or if the data simply reflects random collisions of small recycling endosomes with the enlarged early endosomes. To determine if these collisions are functionally significant the authors would need to show data on the transfer of recycling cargo from Rab5 endosomes to Rab11 endosomes during such interactions. Without this I would recommend removing this data from the paper. Along these lines, while the Snx1 data was more convincing, it was overinterpreted as "recycling" or "sorting" even though no cargo was followed. The authors should more carefully word the text, especially the abstract and discussion, to avoid such gross overinterpretation of the Snx1 data.

We provide now more evidence for the interaction of Rab11 vesicles with the enlarged endosomes. We made videos with shorter intervals between the individual frames (Video Figure 8A supplement 2 and 3). These data clearly show that this is not an accidental bumping into an endosome but that Rab11 vesicles can circle around endosomes and stay for several minutes.

In addition, we imaged TfR-GFP together with mApple-Rab5. These data show that TfR-GFP positive vesicles bud off from mApple-Rab5 positive endosomes and that the GFP fluorescence intensity goes down over time in enlarged endosomes (Figure 9A). These data are consistent with recycling of TfR to the plasma membrane.

Moreover, CDMPR-GFP, which cycles between the TGN and endosomes was found to be present on Rab5 negative enlarged structure, which then turned Rab5 positive, and subsequently lost the CDMPR signal (Figure 9B). Importantly those endosomes could regain CDMPR, which we interpret as acquisition from the TGN (Figure 9, figure supplement 1). These data may indicate that the TGN-endosome shuttle is intact after nigericin washout.

That the TfR and CDMPR are really transported out of the enlarged endosome is also supported by our finding that GalT-GFP stayed in the enlarged endosome and the signal intensity did not significantly drop (Figure 9C).

The dependency of cargo trafficking on Rab11 and Snx1 could not be shown most likely because of the redundancy of these recycling pathways, with alternative pathways compensating for any deficiency in Rab11a or Snx1 (possibly including Rab11b, Rab25, Snx4, Snx8). Therefore, we followed the advice of the Reviewer 2 to be more careful with the wording in order to avoid overinterpretation of Snx1 and Rab11 data.

Regardless of the recycling pathway or the destination of the recycled cargo, these data show that sorting and recycling appear to be functional in the enlarged endosomes. We have not investigated whether TfR or CDMPR is exported away from the endosome via Snx1-coated tubules or Rab11-mediated transport carriers, as this would require triple transfection with BFP-Rab5, which cells do not tolerate well.

(2) To really establish this endosome maturation model the authors should establish if the enlarged endosomes contain endocytosed cargo, as opposed to Golgi-derived cargo, and determine how long it takes to acquire such cargo. This could be accomplished using Tf, EGF, or perhaps dextran at early timepoints after nigericin washout.

As pointed out above, we show now that TfR-GFP is present in enlarged endosomes and is lost from these endosomes over time (Figure 9A,D,G). Moreover, we performed experiments with dextran-Alexa647 and mCherry-nanobodies directed against GFP to show that endocytosed material from the plasma membrane indeed reached the enlarged endosomes (Figure 3 figure supplement 1 and 2). TfR-GFP recruitment to the Rab5-positive endosome coincided with the recruitment of mCherry-tagged anti-GFP nanobody-labeled surface transferrin, suggesting that most of TfR-GFP at the enlarged endosome did indeed come from the plasma membrane. In contrast, CDMPR-GFP is present at the enlarged compartments prior to Rab5 recruitment and is promptly removed during or shortly before Rab5 recruitment (Figure 9B,E,H). These observations suggest that the maturing endosomes readily accept endocytosed cargo and actively remove Golgi-derived cargo. We observed that endocytosed material reached the enlarged endosomes as they became Rab5 positive (Figure 3, figure supplement 1 and 2).

(3) Figure 7 – It was not convincing that data in panels F and G are different from each other.

We agree with the reviewer that the difference between the data presented in panel F and G is not very big. These panels represent the average of many endosomes and with the averaging the differences from the individual traces get cancelled out. The process is asynchronous and thus in this case the individual traces are more telling than the averaged traces. Nevertheless, we decided to keep the average traces in the manuscript because the highlight the asynchronous nature of the process. We modified the text to make this point clear.

(4) Figure 11 – I find the interpretation of panel D confusing. How can we interpret this as connected to Rab conversion when even the labeled compartments at the earliest time point in the czz1 knockout have abnormally high pH, and during the time-course even the last timepoint for czz1 KO is higher than that of the earliest timepoint for WT?

We agree that the ccz1 KO cells display higher endosomal pH than WT cells throughout the time-course. However, the cells in which we express the rescue plasmid of Ccz1 also have apparently less acidified endosomes, even though Ccz1 can still drive Rab conversion, and the pH dropped at an intermediate rate, when comparing rescued cells to control and ccz1 KO cells. Even in ccz1 KO cells endosomal traffic down the degradation pathway is not completely blocked, similarly to what we observed for sand-1 (-/-) *C. elegans* and Mon1a/b knockdown in mammalian cells (Poteryaev et al., 2010). Acidification eventually will occur, but it is massively slowed down; the molecular basis of which is still under investigation in our lab. Even in ccz1 KO cells endosomal traffic down the degradation pathway is not completely blocked, similarly to what we observed for *sand-1 (-/-) C. elegans* and Mon1a/b knockdown in mammalian cells (Poteryaev et al., 2010). Acidification eventually will occur, but it is massively slowed down; the molecular basis of which is still under investigation in our lab.

(5) Figure 12 – The criteria used to determine which GalT structures are Golgi or lysosomes seems flawed. Morphology alone is not sufficient to identify the compartments with high accuracy, especially after perturbation. Also, to what extent does GalT-CFP label lysosomes without nigericin treatment?

To address these issues, we co-labelled cells with lysotracker. GalT-CFP (pHlemon) and lysotracker showed a very high degree of co-localization. These data are included in the manuscript (Figure 10 B).

Reviewer #3 (Recommendations for the authors):

– What is the status of recycling endosomes? These structures must have some relationship to maturing endosomes but the literature is confusing. Rab11 is the traditional marker for recycling endosomes, and the authors describe Rab11 vesicles that "contact" maturing endosomes. How is this pathway envisioned to occur? Does internalized material ever accumulate in Rab11-positive recycling endosomes? It would be useful to bring the earlier work on the FERARI complex into a model that incorporates the current data.

We would predict that the Rab11 vesicles would undergo kiss-and-run on the enlarged endosomes, similarly to what we have described previously. We added a sentence to this effect in the discussion. In fact, we compared Rab11 in control and FERARI KO cells, and it seems as if we indeed see a difference in residence time. However, we consider these experiments and the additional work necessary to make a strong case would be way beyond the scope of this manuscript. Therefore, we included wording in the discussion to integrate our FERARI findings.

– Do late endosomes fuse completely with lysosomes, or do they typically undergo kiss-and-run fusion as has been described in both mammalian and yeast cells? Some of the images here seem to argue against complete fusion. What are the implications? Specifically, if late endosomes are constantly being generated by maturation but are not consumed by fusion with lysosomes, why doesn't the cell fill up with late endosomes?

This is really difficult to distinguish at this point. We think that we observe both events. On one hand we see lysosomes contacting and ‘dancing around’ the enlarged endosomes. On the other hand, we also see them disappearing after contacting endosomes, consistent with a fusion event. At any rate, the enlarged endosomes acidify further and become lysotracker positive. At which point, they will lose their round and becomes smaller over time. In a way the endolysosome matures into a lysosome. We surmise that also membrane recycling will occur, but this is a subject for future studies. We do not observe an accumulation of late endosomes because they turn into endolysosomes and then mature to lysosomes, which then can go on and fuse with late endosomes again. Our data in new Figure 10 figure supplement 1 supports this model.

– The most striking result is that TGN-derived compartments go on to become Rab5-labeled endosomes, yet this topic is not even mentioned in the Discussion. Do the authors infer that there is a close relationship between the TGN and early endosomes, as has been observed in plant and yeast cells? Or is it more likely that existing Rab5-positive early endosomes become more prominent due to fusion with enlarged TGN-derived compartments?

We now included a discussion about TGN-derived compartments becoming Rab5-positive endosomes. We speculate that the Rab5 identity is mostly brought about through fusion with Rab5 positive endocytic structures, as we frequently observe such events. However, we cannot exclude other possible mechanisms such as direct recruitment of Rab5 from the cytoplasm. These alternative or additional mechanisms may just be much harder to recognize and to interpret, especially if occurring in combination.

In Figure 1D, homotypic fusion is readily apparent but I don't see the Rab conversion described in the legend.

We agree with the reviewer that in the particular example Rab conversion is more difficult to detect as the Rab7 signal at the endosome is very weak. However, in Figure 1C this is easy to see. Therefore, we do not refer to panel D anymore when describing Rab conversion. In addition we provide more examples of Rab conversion throughout the manuscript.

The videos are hard to match to the figures. For example, the "Figure 1C supplement 1" video seems to have nothing to do with Figure 1C, and I couldn't find a description of this video.

We apologize for the mistake. This was apparently a mistake introduced by the renaming of all files. We provide now the correct file names. We further integrate the links to all the videos in the figure legends, as the descriptions are identical to the time lapse image series provided in the figures.

Figure 5: PI(3)P is presumably generated, not recruited.

Of course, the reviewer is right. We changed the text accordingly.
